# Development of Functional Composite Cu(II)-Polyoxometalate/PLA with Antimicrobial Properties

**DOI:** 10.3390/molecules27082510

**Published:** 2022-04-13

**Authors:** Ella Duvanova, Illia Krasnou, Andres Krumme, Valdek Mikli, Serhii Radio, Georgiy M. Rozantsev, Yevgen Karpichev

**Affiliations:** 1 Department of Chemistry and Biotechnology, Tallinn University of Technology (TalTech), 12618 Tallinn, Estonia; e.ivantsova@donnu.edu.ua; 2 Research Laboratory “Chemistry of Polyoxometalates and Complex Oxide Systems”, Vasyl’ Stus Donetsk National University, 21027 Vinnytsia, Ukraine; radio@donnu.edu.ua (S.R.); g.rozantsev@donnu.edu.ua (G.M.R.); 3 Department of Materials and Environmental Technology, Tallinn University of Technology (TalTech), 19086 Tallinn, Estonia; illia.krasnou@taltech.ee (I.K.); andres.krumme@taltech.ee (A.K.); valdek.mikli@taltech.ee (V.M.)

**Keywords:** polyoxometalate, copper(II) paratungstate B, poly(lactic acid), antimicrobial surfaces, composite films

## Abstract

Novel composite self-disinfecting films of polylactic acid (PLA) filled with nanosized particles of double sodium–copper(II) paratungstate B Na_2_Cu_3_(CuOH)_2_[W_12_O_40_(OH)_2_]·32H_2_O (POM) were developed. The solvent casting (POM/PLA film) and solvent-free melt extrusion methods (Extr. POM/PLA film) were applied for film preparation. The copper (II) ion release to water from both types of the films after 10 days at different temperatures demonstrated that the PLA matrix acts as a diffusion barrier, and the resulting concentration of released copper in water at room temperature remained low, at 0.79% for POM/PLA film and 0.51% for Extr. POM/PLA film. The POM-containing films reveals a significant inhibitory effect against *E. coli* ATCC 25922 in the agar diffusion test. The numbers of CFUs in washes of the films after incubation for 24 h were found to be 3.6 *log* CFU mL^–1^ (POM/PLA film) and 4.1 *log* CFU mL^–1^ (Extr. POM/PLA film). The films combine the antibacterial properties of POM and a bio-based polymer matrix, which makes them a prospective coating material for applications in hospital indoor environments. Excellent thermal stability of POM gives a technological advantage for industrial manufacturing to allow the processing of novel composite material in the solvent free (molten) state.

## 1. Introduction

Copper is an inexpensive, earth-abundant metal with moderate toxicity and extremely broad use as a green and sustainable alternative for numerous metal-catalyzed organic reactions [1,2]. Copper is well recognized as having antimicrobial activity, but it has not been applied widely to the clinical setting [3], including health care-associated infections, which pose a real danger nowadays. These infections occur while receiving medical or surgical care, or in any other conditions related to treatment. Despite the efforts of environmental and infection control specialists, infections acquired during hospitalization are still one of the leading causes of morbidity and mortality in hospitals around the world. The development of antimicrobial surfaces or coatings offers an opportunity to reduce the risk of infection associated with poor hygiene and ineffective room cleaning [4]. Self-cleaning or self-disinfecting antimicrobial surfaces were developed and put into use only 50 years ago to complement existing infection control methods. The self-disinfecting surfaces proposed in 1964 by Kingston and Noble were developed based on the hypothesis that self-disinfecting solutions reduce the spread of germs as a result of the suppression of bacteria growth during its deposition on the surface in the anthropogenic environment [5]. All the above mentioned self-disinfecting materials can be divided into two main categories, namely anti-adhesive/anti-fouling (polyethylene glycol; polyethylene terephthalate films, modified through the addition of polyethylene oxide) and antimicrobial, either by inhibiting the growth of bacteria or by directly killing the microorganisms (triclosan, silver and copper surfaces) [4]. Thus, the creation of new antibacterial coatings based on soluble and thermoplastic polymeric materials is an urgent task. One of the promising sustainable plastics is polylactic acid (PLA), an inexpensive and bio-based material [6,7,8,9,10,11,12,13]. Table 1 shows data on the improvement of the antibacterial properties of PLA composites by introducing additives with antibacterial properties. Designing new polymeric material with antibacterial properties and the ability to form thin films is a challenging but prospective issue for various biomedical applications.

Special attention should be paid to enhancing the antibacterial properties of PLA with inorganic molecules, e.g., copper(II)-containing polyxometalates. These compounds compare favorably to other antibacterial agents in terms of thermal stability, low solubility in water, and insolubility in organic solvents, as well as ease of preparation from aqueous solutions. Polyoxometalates (POM) is a large class of polyoxoanions formed by the metals of V and VI groups of the Periodic Table with broad application in medicine [14,15,16]. Thus, these compounds can exhibit antitumor activity [17], but their antibacterial properties also deserve great attention. The antibacterial activity of pure inorganic POMs can be divided into two main types, synergistic and direct. Many inorganic POMs did not show significant antibacterial activity at pharmacologically acceptable concentrations but showed synergistic activity with conventional antibiotics. For example, polyoxotungstates had a synergistic effect in combination with *β*-lactam antibiotics on MR/VR *S. aureus*^(+)^ [18,19,20,21,22]; MR *S. epidermis*^(+)^, MR *S. auricularis*^(+)^, and MS *S. haemolyticus*^(+)^ [23]; and MZR *H. pylori*^(−)^ and CLR *H. pylori*^(−)^ [22]. Only a few POMs had direct antibacterial activity [16,18,19,24,25,26,27] tested against various strains of *S. aureus* to show significant antibacterial activity, demonstrating a minimum inhibitory concentration (MIC) < 100 mg mL^−1^, while the effective antibiotics had MIC values of 0.001–10 mg mL^–1^. In the review of Bijelic et al. [16], there was only one copper(II)-containing polyoxotungstate K_12_[Cu_3_(PW_9_O_34_)_2_] mentioned as having MIC values of 400 mg mL^–1^ and 200 mg mL^–1^ against MRSA strains SR3605 and ATCC43300, respectively. In combination with oxacillin, this POM was reported to have FIC values of 0.141 and 0.070 towards the MRSA strains SR3605 and ATCC43300, respectively.

In this study, we report PLA composites with double sodium–copper(II) paratungstate B possessing antibacterial properties and characterized by low copper (II) ion release. The films were prepared by two methods, namely the solvent casting method and the melt extrusion method. The comprehensive characterization of the obtained composite films included determination of physical properties as well as release of Cu(II) ions in water. The surface morphology of the films was studied by scanning electron microscopy (SEM) to demonstrate encapsulation of POM particles inside the polymer matrix, which prevents active leaching of metals upon contact with aqueous environments.

The antibacterial activity of Cu(II)-containing paratungstates B was studied against *E. coli*. The double sodium–copper(II) paratungstate B Na_2_Cu_3_(CuOH)_2_[W_12_O_40_(OH)_2_]·32H_2_O revealed stability, and its structure was reliably determined and retained during the change of the number of water molecules, some of which were uncoordinated and located in the structure channels [28,29]. Na_2_Cu_3_(CuOH)_2_[W_12_O_40_(OH)_2_]·nH_2_O (*n* = 32, 34) nanoparticles can be prepared by an inexpensive low energy consuming method. Its synthesis occurs either as a result of self-assembly in an acidified aqueous solution of Cu^2+^ and WO_4_^2−^ [28,29], or as a result of the hydrolytic transformation of ammonium metatungstate (NH_4_)_6_[W_12_O_38_(OH)_2_]∙3H_2_O in NaOH solution. An additional advantage for choosing this POM has previously been reported as an inhibitory effect of Na_2_Cu_3_(CuOH)_2_[W_12_O_40_(OH)_2_]·nH_2_O against cancer cell lines: human cervical carcinoma HeLa cells (IC_50_ 12.3 μmol·L^−1^), ovarian carcinoma SKOV-3 cells (IC_50_ 43.1 μmol·L^−1^), hepatoma HepG2 cells (IC_50_ 34.2 μmol·L^−1^), and neuroblastoma SHY5Y cells (IC_50_ 100.5 μmol·L^−1^) [17].

## 2. Results and Discussion

### 2.1. Characterization of POM

The POM salts used in the research have a well-established crystalline structure: triclinic, space group *P*–1, a = 10.6836(4) Å, b = 12.9066(6) Å, c = 13.6475(5) Å, α = 73.561(4)°, β = 75.685(3)°, γ = 67.666(4)°, V = 1648.68(12) Å^3^ at T = 293 K [28].

**Figure 1:**
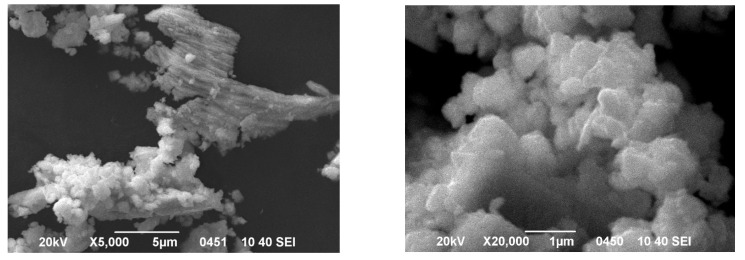
SEM image of POM-powder surface in secondary electron mode: (**left**) ×5000; (**right**) ×20,000.

**Figure 2:**
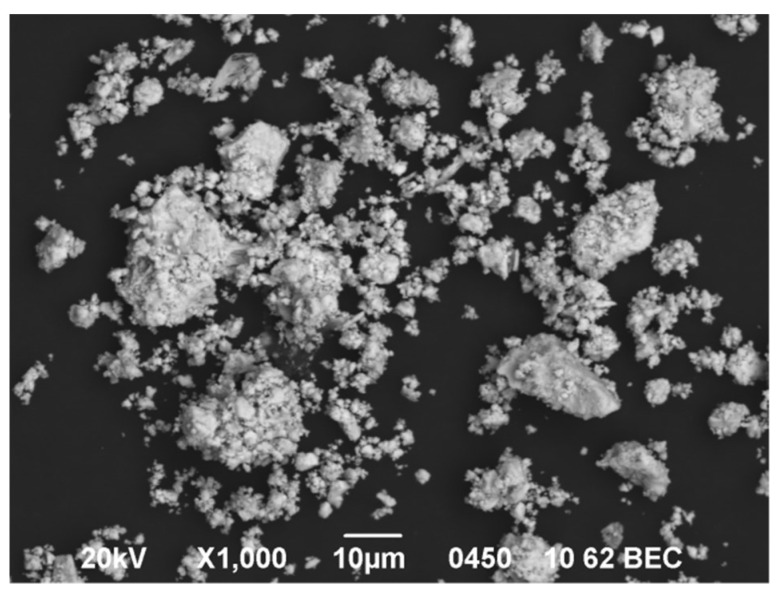
SEM image of POM powder surface in backscattered electron mode.

Microscopic analysis showed that the POM grain surface has fuzzy blurred edges. The grain size of the sample was within the range of 200–400 nm (Figure 1). Uniform surface contrast in BEC mode pointed to the single-phase of the obtained salt (Figure 2). On the micrographs of POM powder in characteristic X-ray emission, there were no regions with different surface morphologies, and there was an even distribution of Na, Cu, W, and O without segregation and liquation (apparent heterogeneities are explained by different relief of the sample surface); see Figure S1. These clearly indicate the formation of a single-phase sample.

X-ray spectral microanalysis was carried out in various areas of the powder surface. The results of elemental analysis presented in Figure S2 show an identical theoretical molar ratio of elements Na:Cu:W = 2.00:5.00:12.00.

The FTIR spectra of POM is shown in Figure S3a.

### 2.2. Preparation and Characterization of PLA Composite Films

**Figure 3:**
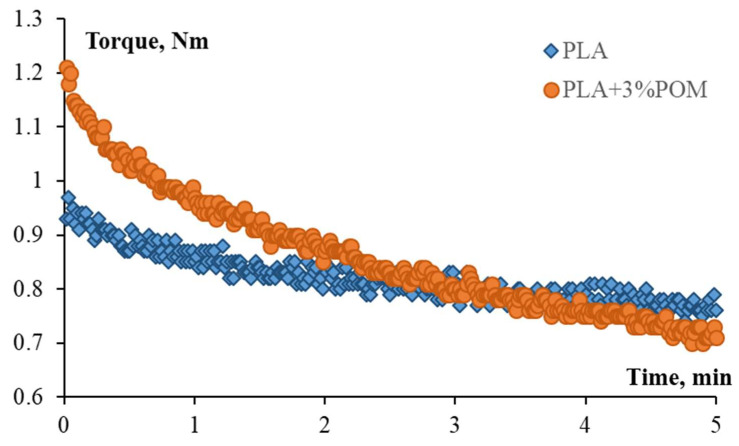
Evolution of torque over time during compounding of Exr. PLA and Extr. POM/PLA materials.

Two types of composite films were successfully obtained by (i) solvent casting (POM/PLA film) and (ii) melt extrusion (Extr. POM/PLA film) methods. Figure 3 represents evolution of torque during compounding. As could be seen, the addition of POM particles to PLA reduced the viscosity of the compound after 3 min of blending. This time was assumed to be a homogenization time, taking into account that significantly longer time may cause thermal degradation of the polymer matrix. After 5 min of compounding, the material extruded out as a round-shaped filament and was collected. For further studies, the filaments were molded into 1 mm sheets by hot-pressing between steel plates with Teflon spacers. Specimens of Extr. PLA appeared transparent with a slightly yellowish color, and Extr. POM/PLA compounds were opaque and yellowish.

**Figure 4:**
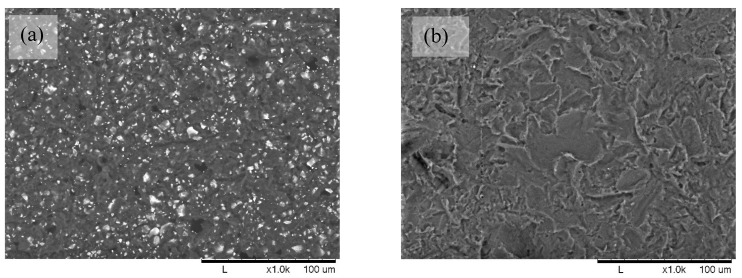
SEM image of solvent casted films: (**a**) POM/PLA film; (**b**) blank PLA film at 1000x magnification.

**Figure 5:**
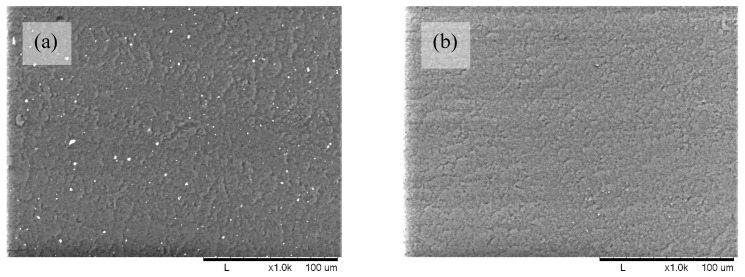
SEM images of extruded films: (**a**) Extr. POM/PLA film; (**b**) blank Extr. PLA film at ×1000 magnification.

The morphology of the films was studied by SEM. The micrograph of POM/PLA film (see Figure 4a) shows many white spots that are evenly distributed over the surface of the PLA material, and there are no large-scale agglomerations. The sizes of the spot ranged from 0.45 to 2.68 µm (Table 2), which allowed us to identify them as POM particles, as shown in Figure 1. Any similar white spots on the micrograph of blank PLA film (see Figure 4b) were detected. POM particles on the surface of Extr. POM/PLA film (see Figure 5a) were different from the particles on the POM/PLA film, as shown in Figure 4a, by smaller size (0.18–1.98 µm, Table 2), as well as a lesser quantity. It can be assumed that extrusion compounding breaks the agglomerates of the POM particles. A comparison of Figure 4a,b and Figure 5a,b shows that the film obtained by the extrusion method is smoother than the film from the solvent casting method. Additionally, it can be seen that POM particles sedimented from solution during film drying, and thus in the bottom side of the film, the amount of POM was larger than in the top side. Both sides of extruded films had very similar amounts of particles because the rapid solidification of the melt prevented particle sedimentation.

**Figure 6:**
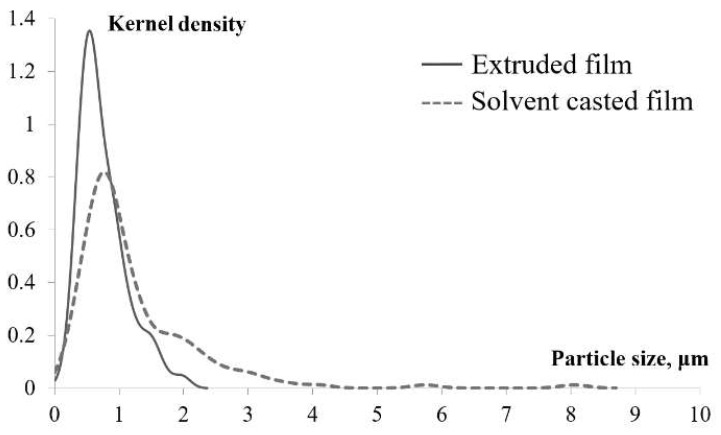
POM particle size distribution in solvent casted (dashed line) and extruded films (solid line).

The particle distribution in the surface of the films was analyzed and is represented in Figure 6. As can be seen, particles up to 9 μm in size were present in solvent casted film, whereas particles in extruded film were limited to 2 μm. Even more, there was a strong tendency for narrowing of the particle size distribution and shifting the majority of particle sizes around 530 nm in extruded films. Solvent casted films had very broad size distribution, with the majority of particles around 750 nm.

Several characteristics of composite films are summarized in Table 2.

#### 2.2.1. FT-IR of Characterization of PLA Composite Films

ATR-FTIR spectra of the POM, two types of blank PLA film, and two POM/PLA-containing film are presented in Figure S3a–e. The main difference between the spectra of both types of POM-containing PLA film and blank PLA film is the presence of absorption bands in the range of 400–960 cm^–1^ (Figure S3b,d), which can be evidence of the paratungstate B anion presence (Figure S3a) in the composition of the films. The positions of absorption peaks of stretching vibrations in the O–W–O framework in the FTIR spectra (for Na_2_Cu_3_(CuOH)_2_[W_12_O_40_(OH)_2_]·32H_2_O (Figure S3a) in cm^–1^ are 506, 576, 673, 798, and 822 (ν_s, as_(O–W–O)); 949 cm^–1^ (ν(W = O_t_)); for PLA–POM film) (Figure S3b) in cm^–1^ are 514, 564, 695, 759, and 829 (ν_s, as_(O–W–O)); 949 cm^–1^ (ν(W = O_t_)); for Extr. PLA–POM film (Figure S3d) in cm^–1^ are 516, 562, 698, 757, and 826 (ν_s, as_(O–W–O)); and 957 cm^–1^ (ν(W = O_t_))) agree well with those determined previously for the salts with the paratungstate B anion [28,29,30]. The band attributed to stretching vibrations of C = O is located at 1748–1749 cm^–1^ for all types of PLA film (Figure S3b–e), which is slightly shifted [10]. The superimposition of the ATR-FTIR spectra are shown on Figure S4. Differences between the spectra of blank PLA film and POM/PLA-containing film can also be seen in the 1050–1250 cm^–1^ range, attributed to C–O and C–O–C stretching vibrations.

#### 2.2.2. Thermal Properties

The thermal behavior of the different POM/PLA composite films was studied by DSC to evaluate how the introduction of POM particles can affect the glass transition (T_g_) and melting (T_m_) temperatures and crystallization parameters of PLA. The addition of the POM filler to PLA resulted in decreasing melting point (T_m_), as shown in Table 3 for both solvent cast and extruded films: by 4 °C for PLA/POM, and by 7 °C for Extr. POM/PLA. The fact of T_m_ depression is the evidence of better dispersion of POM particles within the PLA matrix during melt compounding, as compared to dispersion in solution. Additionally, in the case of the film developed by the solvent casting method (PLA/POM), the T_g_ value increased with addition of POM, and in the case of the Extr. POM/PLA film, the T_g_ value decreased with the addition of POM; see Table 3. Additionally, melting points of extruded films were higher than the solvent cast, which was due to differences in the degree of crystallinity. PLA usually consists of α and α’ crystalline phase mixture, but the casting of the film from a melt at 120 °C transferred all α phase to α’ [31]. Relatively low total crystallinity and low T_g_ allowed uniform thin films to be produced by hot-press or roll-mills. The DSC thermogram is shown in Figure S5.

### 2.3. Copper(II) Ion Release Studies

#### 2.3.1. Copper(II) Ion Release from Air-Dried POM

In order to determine the influence of PLA films on Cu(II) ion release, and air-dried Na_2_Cu_3_(CuOH)_2_[W_12_O_40_(OH)_2_]·32H_2_O sample was investigated first, using 0.020 mg weights in Milli-Q water (10 cm^3^) at room temperature and 40 ± 1 °C. The particles were initially 1.12 ± 0.20 μm in diameter, and after 10 days in Milli-Q water, the size did not change significantly (1.20 ± 0.34 μm). Released total Cu(II) ion concentrations in Milli-Q water were low, amounting to 42.49 ± 0.53 μM and 47.21 ± 0.27 μM at room temperature and 40 ± 1 °C, respectively. This indicates that 1.59 ± 0.02% and 1.79 ± 0.01% by weight of the original POM Na_2_Cu_3_(CuOH)_2_[W_12_O_40_(OH)_2_]·32H_2_O were released into solution.

#### 2.3.2. Copper(II) Ion Release from PLA Composite Films

The mixture of PLA (0.3311 g) and POM (0.0102 g, 3% *w*/*w*) was used for the film preparation by the solvent casting method. The copper content in POM was 8.24% *w*/*w*, which corresponds to 0.8 mg. Therefore, the films contained 0.25 w% of copper (to the total weight of the mixture). The copper content in the film obtained by extrusion was also 0.25% *w*/*w* in the case of 3% *w*/*w* of the POM, which was used to preparation of compound. The copper ion release value was sown on the basis of the POM weight (8.24% *w*/*w*) for convenience. Over 10 days of copper release from two types of PLA composite films in Milli-Q water at room temperature and 40 ± 1 °C, the film size, weight, and thickness did not change significantly (Table 2 and Table 4). In the case of Extr. PLA composite film, released total Cu(II) ion concentrations in Milli-Q water were lower than in Na_2_Cu_3_(CuOH)_2_[W_12_O_40_(OH)_2_]·32H_2_O itself, namely 4.09 ± 0.27 μM and 4.56 ± 0.22 μM at room temperature and 40 ± 1 °C, respectively. This amounts to 0.51 ± 0.03% and 0.57 ± 0.03% of the total Cu(II) content. In its turn, released total Cu(II) ion concentrations in Milli-Q water for PLA composite film was found to be 1.73 ± 0.15 μM (0.79 ± 0.07%) and 1.89 ± 0.20 μM (0.86 ± 0.09%) at room temperature and 40 ± 1 °C, respectively.

Therefore, the novel POM/PLA composite is characterized by remarkably low release of copper ions to the environment.

### 2.4. Antimicrobial Properties of POM and PLA Composite Films

**Figure 7:**
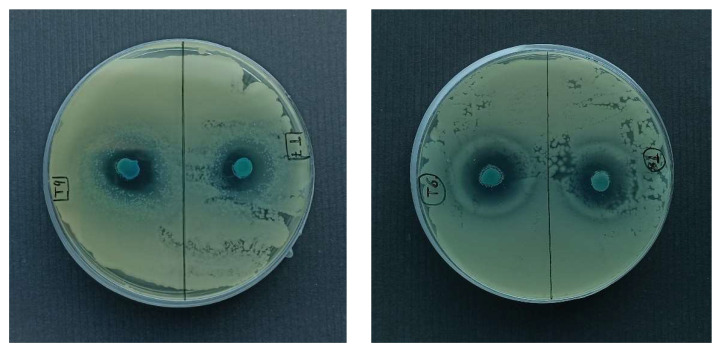
POM agar diffusion test against *E. coli*: Na_2_Cu_4_[W_12_O_40_(OH)_2_]·22H_2_O (T6), Cu_5_[W_12_O_40_(OH)_2_]·30H_2_O (T7), Cu_5_[W_12_O_40_(OH)_2_]·2Cu(OH)_2_·30H_2_O (T8), Na_2_Cu_3_(CuOH)_2_[W_12_O_40_(OH)_2_]·32H_2_O (T9).

After incubation, the antibacterial activity of POMs (Table 5) was evaluated by measurements of the inhibition zone width, see Figure 7.

The POM Na_2_Cu_3_(CuOH)_2_[W_12_O_40_(OH)_2_]·32H_2_O (T9) showed the largest inhibition zone diameter (see Table 5) and was chosen as antimicrobial filler for PLA films. Along with this, Na_2_Cu_3_(CuOH)_2_[W_12_O_40_(OH)_2_]·32H_2_O in suspension showed activity against *E. coli*, with a MIC of 250 µg mL^−1^. The Cu^2+^ release at room temperature from POM determined in Section 2.3.1. was 1.59 ± 0.02%. Thus, the calculated MIC(Cu^2+^) = 3.9 µg mL^−1^. Along with this, the antimicrobial effect of copper(II) sulfate against *E. coli* was studied in [32]; MIC (CuSO_4_·5H_2_O) = 18.8 µg mL^−1^, where the value MIC(Cu^2+^) = 3.0 µg mL^−1^ was calculated, which is almost the same as the value MIC(Cu^2+^) reported in this study. These results allowed us to assume that POM antimicrobial action occurs due to the presence of copper(II) ions. Additionally the MBC value of 750 µg·mL^−1^ was determined (Figure S6), which was not much higher than the MBCs of nanoparticulate metals, and metal oxides against *E. coli* (500 µg·mL^−1^ of Cu_2_O; 250 µg·mL^−1^ of CuO; 250 µg·mL^−1^ of Cu), mentioned in [33], could be the evidence of antimicrobial activity.

**Figure 8:**
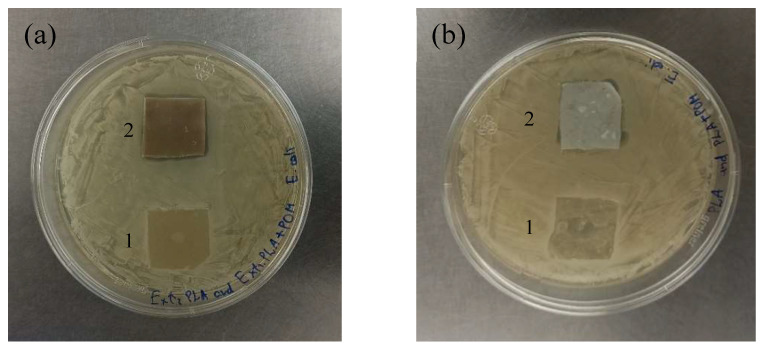
Agar diffusion test for *E. coli* showed the inhibition zone presence: (**a**) Films obtained by electrostatic extrusion method; the control blank Extr. PLA film is marked with 1, while the Extr. POM/PLA composite film is marked with 2. (**b**) Films obtained by solvent casting method; the control PLA film is marked with 1, while the POM/PLA composite film is marked with 2.

The agar diffusion method was reported as a useful method in testing antimicrobial properties of specific polymer materials with antimicrobial adding aimed at creating antimicrobial coatings [9,34]. Figure 8 shows the agar diffusion tests after 24 h against *E. coli* for POM/PLA composite films, where blank PLA films served as a control. Control films did not induce any inhibition zone in either bacterial strain, while the POM/PLA composite films formed an inhibition zone of about 1–2 mm. The inhibition zone was not uniform due to distribution of Cu(II) paratungstate B microbeads within the films and, thus, nonuniform copper release.

Polymer composites showed the presence of an inhibition zone, so the number of CFU·mL^−1^ was counted after the inoculation of the films. The antibacterial activity of POM/PLA films was compared with a control polymer matrix [9]. The number of CFUs within the resulting suspensions was counted after 24 h of incubation; see Table 6. Results demonstrated high activity of POM-containing PLA films compared to the control polymer matrix. In particular, a cell loads equal to 4.1 *log* CFU·mL^−1^ was measured at the end of the test period in the Extr. POM/PLA sample (Figure S7a); this value was lower than that measured in the blank Extr. PLA sample (5.4 *log* CFU·mL^−1^; Figure S7b). In the case of films made by the solvent casting method, the POM/PLA sample (Figure S8a) was found to have a cell load equal to 3.6 *log* CFU·mL^−1^; this value was lower than that measured in blank PLA sample (5.8 *log* CFU·mL^−1^; Figure S8b). This experimental result confirms the efficiency of PLA films filled with Cu(II)-containing POMs to prevent the *E. coli* proliferation in novel smart coating.

**Figure 9:**
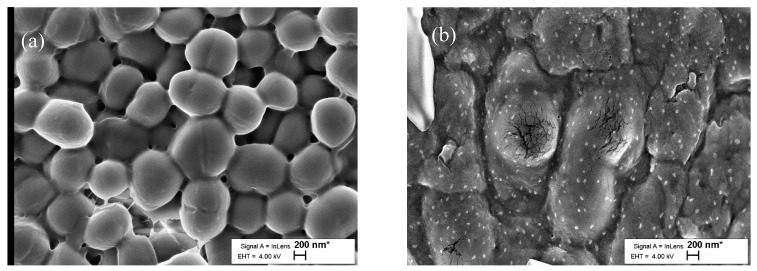
SEM images of *E. coli* biofilm on PLA films surfaces: (**a**) blank PLA film; (**b**) POM/POM film in ×10,000 magnification.

SEM analysis of the biomass on the films (Figure 9a,b) allowed us to observe the morphological changes of the *E. coli* cells during a 24 h period. Healthy cells from blank PLA film had smoother surfaces (Figure 9a), while *E. coli* cells from PLA–POM film had a coarser surface structure, and cell destruction was noticeable; see Figure 9b [35,36].

It can be assumed that the toxic antimicrobial effect of POM/PLA composite films is completely due to the action of POM. Recent studies indicate that the release of metal ions is the driving force of the antimicrobial properties of, in essence, nanoparticles [37,38,39]. A similar approach can be applied for understanding antimicrobial activity of POMs, from which Cu(II) ions can be released. The Cu(II) ion release from the POM/PLA composite films in water is quite low; moreover, at room temperature, its value is 2 (POM/PLA film) and 3 (Extr. POM/PLA film) times less than that from POM itself. At the same time, POM/PLA composite films showed a high value of antibacterial activity. According to the literature data [39,40], the following mechanism may occur in the presence of bacteria: (1) adsorption of bacteria on the polymer surface triggers the diffusion of water through the polymer matrix due to the medium surrounded the bacteria; (2) water with dissolved oxygen reaches the surface of embedded POM particles, allowing dissolution or corrosion processes and in this way Cu(II) ions are realized; (3) Cu(II) ions reach the composite surface, damaging the bacteria membrane; (4) afterwards, Cu(II) ions can diffuse into the bacteria.

## 3. Materials and Methods

### 3.1. Materials

In the study, Na_2_WO_4_ 2H_2_O, HNO_3_, and Cu(NO_3_)_2_ were used as purchased from Sigma-Aldrich without further purification; aqueous solutions were prepared using Milli-Q demineralized water.

Polylactic acid (PLA 3052 D) with a specific gravity of 1.24 g mL^−1^, molecular weight of ca. 1.4 × 10^5^ g mol^−1^, and melt flow index (MFI) of 14 g/10 min (210 °C, 2.16 kg) was supplied by Nature Works^®^, Minnetonka, MN, USA. Medium viscosity sodium alginate (A2033) was supplied by Sigma (St. Louis, MO, USA).

### 3.2. Synthesis of POMs

The polyoxotungstate Na_2_Cu_3_(CuOH)_2_[W_12_O_40_(OH)_2_]·32H_2_O (T9) used in this study was obtained from the Na_2_WO_4_:HNO_3_:Cu(NO_3_)_2_:H_2_O solution according to a reaction described elsewhere [28]. For this purpose, Na_2_WO_4_ (10 mmol) solution was acidified with HNO_3_ to *Z* = ν(HNO_3_)/ν(Na_2_WO_4_) = 1.17 value, and 1.5 molar excess of Cu(NO_3_)_2_ solution was added with vigorous stirring. Immediately after addition of the Cu(NO_3_)_2_ solution, the formation of light blue precipitate was observed. The solution with the precipitate was stirred for 8 h and then left for 3 days at 279 K. Thereafter, the solid amorphous Cu(II) paratungstate B Cu_5_[W_12_O_40_(OH)_2_]·nH_2_O was filtered, and the filtrate was left tightly closed at room temperature. In one week, light blue prismatic crystals of Na_2_Cu_3_(CuOH)_2_ [W_12_O_40_(OH)_2_]·32H_2_O appeared. The crystals were filtered, washed with demineralized water, and left for drying in the air until constant weight was reached, prior to instrumental characterization. The powders of POMs Na_2_Cu_4_[W_12_O_40_(OH)_2_]·22H_2_O (T6), Cu_5_[W_12_O_40_(OH)_2_]·30H_2_O (T7), and Cu_5_[W_12_O_40_(OH)_2_]·2Cu(OH)_2_·30H_2_O (T8) were obtained from the Na_2_WO_4_–HNO_3_–Cu(NO_3_)_2_–H_2_O solution acidified with HNO_3_ to *Z* = ν(HNO_3_)/ν(Na_2_WO_4_) = 1.17 by the procedure described in [30].

### 3.3. Preparation of PLA Composite Films

Prior to film preparation, POM particles were suspended in ethanol and sonicated to break the agglomerates of particles and then dried at room temperature.

#### 3.3.1. The Solvent Casting Method

Two types of films were prepared, namely blank PLA (0.33 g) and the mixture of PLA (0.33 g) and POM (0.01 g); thus, the content of POM in PLA–POM film was 3 wt.%. For this purpose, required amounts of PLA and a mixture of PLA with POM were dissolved in 3 mL of chloroform (10 wt.%) at vigorous stirring. After stirring for 30 min, 0.30 mL of DMSO was added. Addition of DMSO extends the time of solvents evaporation and prevents film from shrinkage during solvent casting. The resulting solution was poured onto a clean glass surface. The resulting films were dried for 12 h at room temperature and then 1 h in a vacuum oven at 50 mbar and 105 °C, which is sufficient to remove the DMSO completely.

#### 3.3.2. The Melt Extrusion Method

PLA granules were milled into a powder by a laboratory knife mill Retsch SM100 with a 1 mm sieve. Next, POM powder was added to PLA and mechanically pre-mixed in a beaker. The POM load was set as 3 wt.% of the compound. Five grams of the pre-mixed blend was loaded into a Haake MiniCTW micro-conical counter-rotating twin-screw extruder at 205 °C, which allowed the thermal stability of PLA to be maintained, and blended for 5 min at 150 rpm via a backflow channel. The compound of pure PLA was prepared under the same conditions. Extruded filament was hot-pressed into a thin film between stainless steel plates at 120 °C and pressure of 70 bar. The low glass transition point of PLA of around 60 °C allowed manufacturing of thin films by hot pressing with no difficulties.

### 3.4. Copper(II) Ion Release Studies

#### 3.4.1. Copper(II) Ion Release Studies from POM

For studying Cu(II), ion release from an air-dried POM sample, 20 ± 1 mg of POM, was added to 10 mL of Milli-Q water. The experiment was performed in a glass vial at room temperature and at 40 ± 1 °C under atmospheric conditions. POM samples were kept in water for 10 days. All experimental points were performed in triplicate. The supernatant was analyzed for Cu(II) content using the ICP-MS method.

#### 3.4.2. Copper(II) Ion Release Studies from Composite PLA Films

Rectangular composite films (2 cm × 3 cm) were immersed in closed glass tubes with 10 mL of Milli-Q water. Total Cu(II) ion release from the films after 10 days at room temperature and 40 ± 1 °C was studied under standard atmospheric conditions. All experimental points were performed in triplicate [9].

### 3.5. Analytical Methods

#### 3.5.1. Scanning Electron Microscopy (SEM)

Field-emission scanning electron microscopy (FE-SEM) was used to analyze cross sections of PLA composite films and to prove the presence of POM particles in the films. The PLA composite films were cross-sectioned, and FE-SEM analyses were performed by using a MIRA 3 XMU field emission scanning electron microscope (Tescan USA Inc., Cranberry Township, PA, USA).

The films’ morphology was studied by means of a tabletop scanning electron microscope TM-1000 Hitachi. The Zeiss EVO MA15 (Carl Zeiss Microscopy GmbH, Jena, Germany) in the secondary electron scattering mode was used for *E. coli* imaging. Particle size distribution was analyzed by Image J freeware (developed by National Institutes of Health USA) and AMC Statistics add-in for MS Excel (developed by the Royal Society of Chemistry).

#### 3.5.2. Fourier Transform Infrared Spectroscopy (FT-IR) of PLA Composite Films

Attenuated total reflectance—Fourier transform infrared (ATR-FTIR) spectroscopy was used to investigate the chemical changes in the PLA matrix due to its potential interactions with POM after thermal processing. Spectra were obtained from two types of blank PLA films and two PLA–POM-containing films using an IRTracer-100 FTIR spectrophotometer (Shimadzu, Kyoto, Japan). This study was conducted in the 400–4000 cm^−1^ range with a 2 cm^−1^ resolution, and number of scans of 40.

#### 3.5.3. Thermal Characterization of PLA Composite Films

Differential scanning calorimetry (DSC) tests were carried out using a Q200 calorimeter (TA Instruments Mod., New Castle, DE, USA). Tests were performed in the temperature range of −25–210 °C at a heating rate of 10 °C min^−1^ for PLA and PLA–POM composite films. The glass transition (T_g_), melting point (T_m_), and enthalpy of fusion (Δ*H*_m_) were measured. Degree of crystallinity was calculated according to the following equation:

(1) $$\chi_{c}=\frac{\Delta H_{m}-\Delta H_{c}}{\omega \Delta H_{m}^{0}}\times 100\%$$

where $\Delta H_{m}$ is the enthalpy of melting, $\Delta H_{c}$ is cold crystallization enthalpy, *ω* is the weight fraction of PLA, and $\Delta H_{m}^{0}$ is melting enthalpy of 100% crystalline PLA (107 J/g).

#### 3.5.4. Mass Spectrometry

An inductively coupled plasma mass spectrometer (ICP-MS) equipped with a hexapole collision cell (PQExCell, Thermo Electron, Winsford, UK) was used to determine the Cu(II) ion content released from POM-containing composite films (see Section 3.4).

### 3.6. Antimicrobial Studies

#### 3.6.1. Antimicrobial Properties of POMs and Determination of MBC

A series of POMs (Na_2_Cu_4_[W_12_O_40_(OH)_2_]·22H_2_O (T6), Cu_5_[W_12_O_40_(OH)_2_]·30H_2_O (T7), Cu_5_[W_12_O_40_(OH)_2_]·2Cu(OH)_2_·30H_2_O (T8), Na_2_Cu_3_(CuOH)_2_[W_12_O_40_(OH)_2_]·32H_2_O (T9)) was tested by the agar diffusion method against the *E. coli* ATCC 25922 strain. The inoculum of bacterial strain was prepared from fresh overnight culture in nutrient broth (NB) (5 g L^−1^ peptone, 3 g L^−1^ meat extract) that were incubated at 37 °C. The agar diffusion test was performed in nutrient agar (NA) (10 g L^−1^ peptone from meat, 10 g L^−1^ meat extract, 5 g L^−1^ NaCl, 18 g L^−1^ agar) and 1% of the bacterial culture in order to obtain 10^5^ CFU·mL^−1^. The diffusion was carried out by pouring 0.1 mL of the media into Petri dishes. The dishes were left for 15 min to dry in air, and the weight of a corresponding POM (~0.05 g) mentioned in Table 5 was placed on the sterile filter-disks on the agar surface and incubated for 24 h at 37 °C. Antibacterial activity was evaluated by measurements of width of the inhibition zone.

The amounts of 2000, 1000, 750, 500, 250, 200, 150, 100, 50, and 20 µg·mL^−1^ of suspended POM were used to determine the minimum inhibitory concentration (MIC) and minimum bactericidal concentration (MBC) required to prevent the growth of bacteria after transferring them onto media. Approximately 2.7 × 10^7^ CFU·mL^−1^ of *E. coli* were added to the POM suspension at a dilution of 1 in 100. Incubation was then carried out in a shaking incubator (200 rpm at 37 °C in air) for 24 h. Inoculated POM-free broth was used as negative controls. Bacteria growth was then assessed by plating each POM/bacterial suspension onto NA plates, followed by the incubation at 37 °C for 24 h [33].

#### 3.6.2. Antimicrobial Properties of PLA Composite Films

Antimicrobial effects of PLA composite films and pure PLA films as a control were investigated by the agar diffusion method. PLA films were prepared as described in Section 3.3 and kept under vacuum for 3 h in order to reach complete vaporization of the traces of chloroform. The resulted films were sterilized for 2 h by means of UV light. The inoculum of bacterial strains was prepared from the fresh overnight culture in NB followed by incubation at 37 °C. The agar diffusion test was performed in 1% of the bacterial culture in order to obtain 10^5^ CFU mL^−1^. The diffusion technique was conducted by pouring 0.2 mL^−1^ of the media into Petri dishes. The dishes were then left for 15 min to dry in air, and film samples were placed on the agar surface to incubate at 37 °C for 24 h. Antibacterial activity was evaluated by measurements of the inhibition zone width [9].

The antibacterial activity of the PLA composite films against *E. coli* was also tested according to Japan Industrial Standard JIS Z 2801:2000 [41,42]. The inoculum of bacterial strains was prepared from fresh overnight culture in NB as described above. Then the strains were centrifuged for 30 min at 5000 rpm and diluted by saline solution (50 mL; 0.9 g of sodium chloride dissolved in 1 L of Milli-Q water). The final concentration of 27 × 10^6^ CFU·mL^−1^ was determined by the serial dilution method. An aliquot of the resulting cell suspension (1 mL) was then placed onto both untreated surfaces and nanostructured films (3 × 3 cm^2^) that were then incubated at 25 °C for 16 h in sterile Petri dishes. After incubation, 5 mL of saline solution were added in each Petri dish. Before sampling, the dishes were shaken for 180 s. The wash was serially diluted, and aliquots of each dilution were spread on NA. The medium was incubated at 37 °C for 24 h. The number of CFUs within the resulting suspensions was enumerated. Data from JIS Z 2801:2000 are usually expressed as an antibacterial value calculated from the difference between the *log* CFU on the treated surface and that measured on the untreated surface, although in some cases, the actual data as CFU cm^−2^ are presented. A number of checks are imposed by JIS Z 2801 to validate the results.

## 4. Conclusions

Novel composite POM/PLA films show strong potential as coatings with antimicrobial activity, convenient for application in hospital indoor environments. POMs have strong advantages over conventional antibiotics due to excellent thermal stability, which makes possible the application of melt extrusion solvent-free methods for the development of the coatings based on thermoplastic polymers. One could conclude that melt extrusion is an efficient and technological approach for antimicrobial surface preparation with uniform distribution of bioactive filler. The antimicrobial activity of Cu(II)-containing polyoxometalates was confirmed by the agar diffusion test. The MIC = 250 µg·mL^−1^ and MBC = 750 µg·mL^−1^ values were found for Na_2_Cu_3_(CuOH)_2_[W_12_O_40_(OH)_2_]·32H_2_O against *E. coli*. The agar diffusion method showed 1–2 mm inhibition zone diameters for both types of films. The values of the antimicrobial activity of films against *E. coli* were determined to be higher for films obtained by the solvent casting method (3.6 *log* CFU·mL^−1^) than for films obtained by the extrusion method (4.1 *log* CFU·mL^−1^).

## Figures and Tables

**Table 1:** Functionalized PLA-based materials: preparation methods and antibacterial activity.

Material Preparation Method	Antimicrobial Agent	Agent Content	Antimicrobial Activity		Ref.
Solvent casting method	Copper modified montmorillonite (MtCu^2+^ and MtCu^0^)	1, 3, and 5 wt.%	MtCu^2+^ against *E. coli*: 1 wt.% 3.68 *log* CFU mL^–1^ (99.97% reduction) 3 wt.% 5.64 *log* CFU mL^–1^ (99.99% reduction) 5 wt.% 5.75 *log* CFU mL^–1^ (99.99% reduction)	MtCu^0^ against *E. coli*: 1 wt.% 1.46 *log* CFU mL^–1^ (96.53% reduction) 3 wt.% 1.76 *log* CFU mL^–1^ (98.28% reduction) 5 wt.% 5.63 *log* CFU mL^–1^ (99.99% reduction)	[8]
	Alginate microbeads containing silver nanoparticles (AgNPs)	5 wt.%	Agar diffusion method results (inhibition zone diameter): 1–2 mm (*E. coli*) and 4 mm (*S. aureus*)		[9]
	Poly lactic-b-4-vinylpyridine (PDLA-b-P4VP); Ag-nanoparticles	arbitrary	PLLA/PDLA_255_-P4VP_46_ against *E. coli*, 83.2% reduction; PLLA/PDLA_255_-P4VP_46, 75, 98_/Ag against *E. coli*, 100% reduction		[10]
	Cu-nanoparticles	0.3 wt.%	Activity against mix of *P. fluorescens* and *P. putida*: 6.0 *log* CFU·mL^–1^ (CuNPs-C-PLA), 7.4 *log* CFU·mL^–1^ (blank PLA)		[11]
Electrospinning method	Fibrillar particles of Ag-sepiolite and Cu-sepiolite	5 wt.%	35% (*P. putida*), 85% (*S. cerevisiae*) reduction		[12]
	Copper salt ion-exchange zeolite nanoparticles (nZH-Cu)	1, 2, and 3 wt.%	PLA 1%, nZH-Cu 55.3% (*S. aureus*), and 62.7% (*S. Typhi*) reduction; PLA ≥2%, nZH-Cu 100% (*S. aureus* and *S. Typhi*) reduction		[13]

**Table 2:** The main characteristics of the POM/PLA composite films.

Sample	Weight (g)	Film Thickness (mm)	POM Particles Average Size (µm)	POM Particle Spacing (µm)
Extr. POM/PLA composite film	0.215 ± 0.001	1.21 ± 0.04	0.75 ± 0.37	17 ± 9
PLA+POM	0.056 ± 0.006	0.17 ± 0.02	1.03 ± 0.48	11 ± 4

**Table 3:** Thermal properties of PLA with POM compounds.

Sample	T_m_, °C	ΔH_m_, J/g	χ_c_, %	T_g_, °C
blank PLA film	154	46.2	43	60
POM/PLA film	150	37.7	36	62
blank Extr. PLA film	171	43.2	40	60
Extr. POM/PLA film	164	37.5	36	59

**Table 4:** The main characteristics of the POM/PLA composite films after 10 days of immersion in Milli-Q water at different temperature.

Sample	Temperature	Weight (g)	Film Thickness (mm)
Extr. POM/PLA	room temperature	0.204 ± 0.001	1.195 ± 0.015
	40 °C	0.207 ± 0.001	1.245 ± 0.029
POM/PLA	room temperature	0.047 ± 0.005	0.156 ± 0.005
	40 °C	0.049 ± 0.002	0.136 ± 0.013

**Table 5:** The results of POM agar diffusion test against *E. coli*.

POM	T6	T7	T8	T9
Weight, g	0.0518	0.0521	0.0520	0.0519
Inhibition zone diameter, mm	14	13	12	16

**Table 6:** The results of *E. coli* CFU counting after inoculation of blank PLA films and Cu(II)-containing POM/PLA films.

Extr. PLA Film	Dilution Factor, D	Number of Colonies	The Number of Viable Cells (CFU·mL^−1^)	PLA Film	Dilution Factor, D	Number of Colonies	The Number of Viable Cells (CFU·mL^−1^)
Blank 1	10^2^	247	(2.45 ± 0.19) × 10^5^	Blank 1	10^3^	59	(6.13 ± 0.59) × 10^5^
Blank 2		225		Blank 2		57	
Blank 3		262		Blank 3		68	
Sample 1	10^1^	121	(1.39 ± 0.22) × 10^4^	Sample 1	10^1^	38	(4.1 ± 0.26) × 10^3^
Sample 2		164		Sample 2		42	
Sample 3		133		Sample 3		43	

## Data Availability

The data presented are available in the manuscript.

## Supplementary Materials

The following materials are available online at https://www.mdpi.com/article/10.3390/molecules27082510/s1: Figure S1: SEM images of Na_2_Cu_3_(CuOH)_2_[W_12_O_40_(OH)_2_]·32H_2_O powder in the characteristic X-ray emission; Figure S2: Surface micromorphology of triturated Na_2_Cu_3_(CuOH)_2_[W_12_O_40_(OH)_2_]·32H_2_O sample; Figure S3: ATR-FTIR spectra: (a) Na_2_Cu_3_(CuOH)_2_[W_12_O_40_(OH)_2_]·32H_2_O, (b) PLA-POM film, (c) blank PLA film, (d) Extr. PLA-POM film, (e) blank Extr. PLA film; Figure S4: The superimposition of the ATR-FTIR spectra; Figure S5: The DSC thermograms; Figure S6: The results of minimum bactericidal concentration (MBC) determination Na_2_Cu_3_(CuOH)_2_[W_12_O_40_(OH)_2_]·32H_2_O; Figure S7: *E. coli* CFU after inoculation of the films in the wash by several dilutions; Figure S8: *E. coli* CFU after inoculation of the PLA-POM film and blank PLA film in the wash by several dilutions. SEM images of POM; ATR-FTIR spectra of POM, DSC thermograms, minimum bactericidal concentration (MBC) determination and E. coli CFU.
