# Supplementary material for: Development of Functional Composite Cu(II)-Polyoxometalate/PLA with Antimicrobial Properties
Source: Molecules. 2022 Apr 13;27(8):2510. doi: 10.3390/molecules27082510 (PMC9025916; doi:10.3390/molecules27082510)

molecules

Electronic Supporting Information

Development of functional composite Cu(II)-polyoxometalate/ PLA with antimicrobial properties

Ella Duvanova[1,2], Illia Krasnou[3], Andres Krumme[3], Valdek Mikli[3], Serhii Radio[2], Georgiy Rozantsev[2], Yevgen Karpichev[1]

[1] Department of Chemistry and Biotechnology, Tallinn University of Technology (TalTech), Estonia

[2] Research Laboratory "Chemistry of Polyoxometalates and Complex Oxide Systems" Vasyl' Stus Donetsk National University, Vinnytsia, Ukraine

[3] Department of Materials and Environmental Technology, Tallinn University of Technology (TalTech), Estonia

Table of Content

Figure S1. SEM images of $Na_2Cu_3(CuOH)_2[W_{12}O_{40}(OH)_2]\cdot 32H_2O$ powder in the characteristic X-ray emission.

Figure S2. Surface micromorphology of triturated $Na_2Cu_3(CuOH)_2[W_{12}O_{40}(OH)_2]\cdot 32H_2O$ sample.

Figure S3. ATR-FTIR spectra: (a) $Na_2Cu_3(CuOH)_2[W_{12}O_{40}(OH)_2]\cdot 32H_2O$, (b) PLA-POM film, (c) blank PLA film, (d) Extr. PLA-POM film, (e) blank Extr. PLA film

Figure S4. The superimposition of the ATR-FTIR spectra: (1) blank PLA film, (2) blank Extr. PLA film, (3) PLA-POM film, (4) Extr. PLA-POM film.

Figure S5. The DSC thermogram: (1) PLA-POM film, (2) blank PLA film, (3) Extr. PLA-POM film, (4) blank Extr. PLA film.

Figure S6. The results of minimum bactericidal concentration (MBC) determination for $Na_2Cu_3(CuOH)_2[W_{12}O_{40}(OH)_2]\cdot 32H_2O$.

Figure S7. *E. coli* CFU after inoculation of the films in the wash by several dilutions: (a) Extr. PLA-POM film, (b) blank Extr. PLA film.

Figure S8. *E. coli* CFU after inoculation of the PLA-POM film and blank PLA film in the wash by several dilutions.

Figure S1. SEM images of $Na_2Cu_3(CuOH)_2[W_{12}O_{40}(OH)_2]\cdot 32H_2O$ powder in the characteristic X-ray emission.

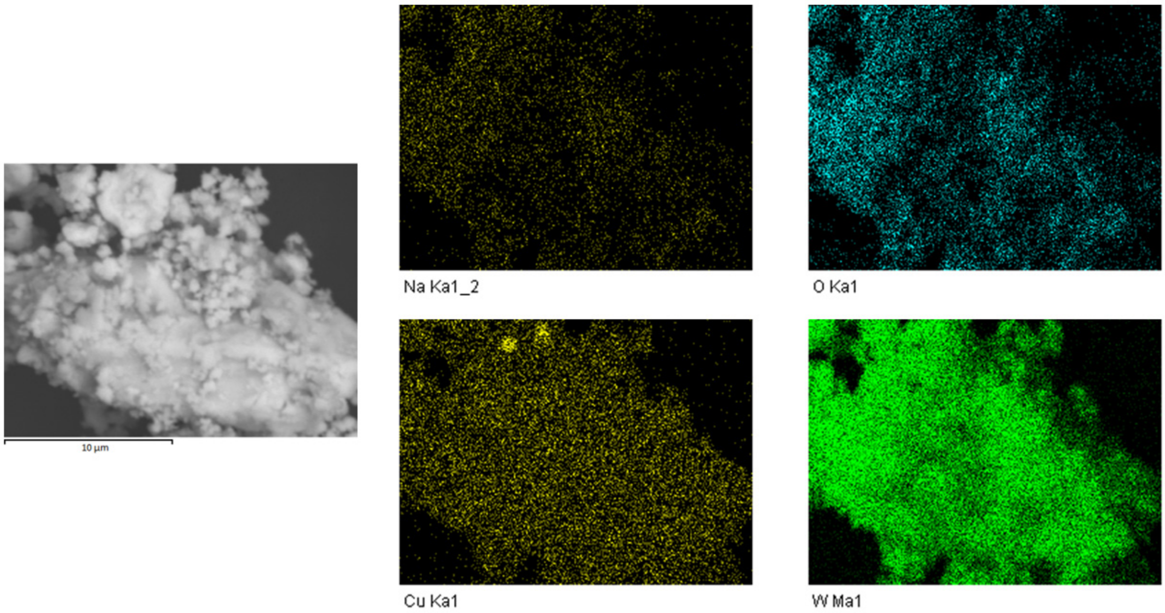

Figure S2. Surface micromorphology of triturated $Na_2Cu_3(CuOH)_2[W_{12}O_{40}(OH)_2]\cdot 32H_2O$ sample. EDX analysis was made in points indicated (see table below).

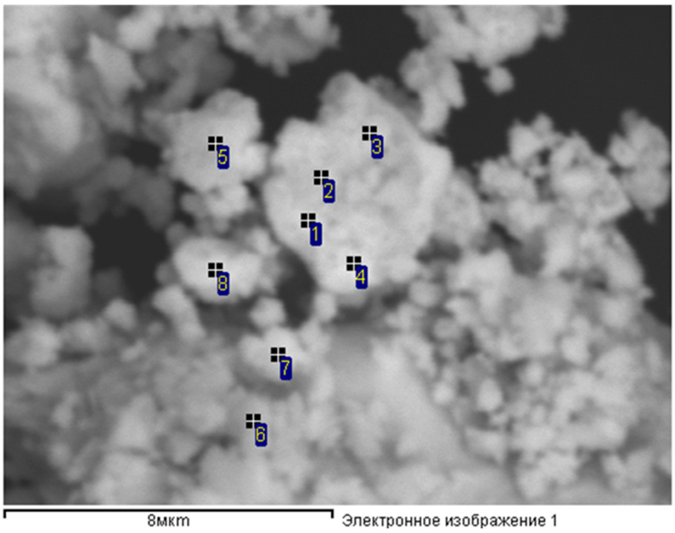

Atomic ratio of Na, Cu, and W in different points marked on Figure SI2 of triturated $Na_2Cu_3(CuOH)_2[W_{12}O_{40}(OH)_2]\cdot 32H_2O$ sample:

at.%	Points								Average	**Atomic ratio**
	**1**	**2**	**3**	**4**	**5**	**6**	**7**	**8**		
Na	1.25	1.19	1.16	1.21	1.20	1.15	1.13	1.18	1.18	1.99
Cu	8.33	8.24	8.20	8.31	8.30	8.27	8.32	8.24	8.28	5.02
W	57.11	57.09	57.20	57.19	57.15	57.25	57.28	57.20	57.18	11.99

Figure S3. ATR-FTIR spectra: (a) $Na_2Cu_3(CuOH)_2[W_{12}O_{40}(OH)_2]\cdot32H_2O$, (b) PLA-POM film, (c) blank PLA film, (d) Extr. PLA-POM film, (e) blank Extr. PLA film

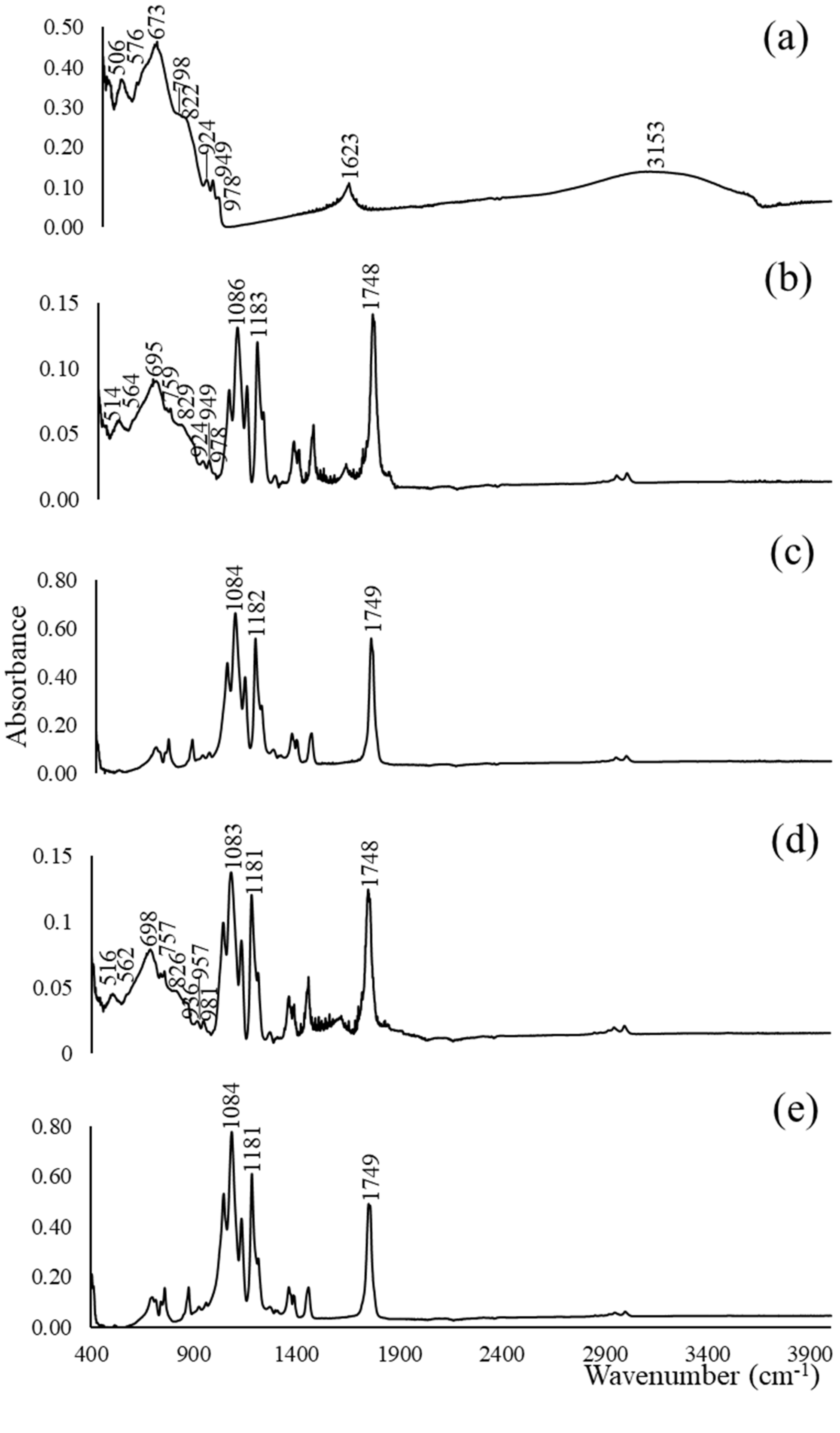

Figure S4. The superimposition of the ATR-FTIR spectra: (1) blank PLA film, (2) blank Extr. PLA film, (3) PLA-POM film, (4) Extr. PLA-POM film.

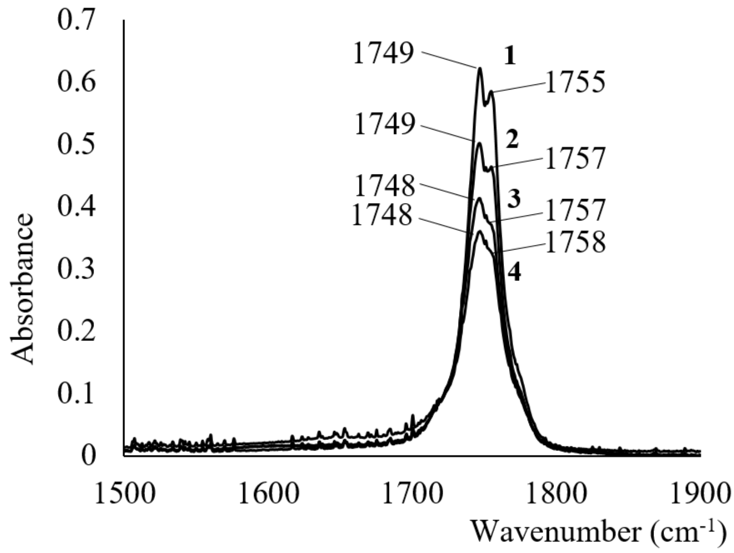

Figure S5. The DSC thermogram: (1) PLA-POM film, (2) blank PLA film, (3) Extr. PLA-POM film, (4) blank Extr. PLA film.

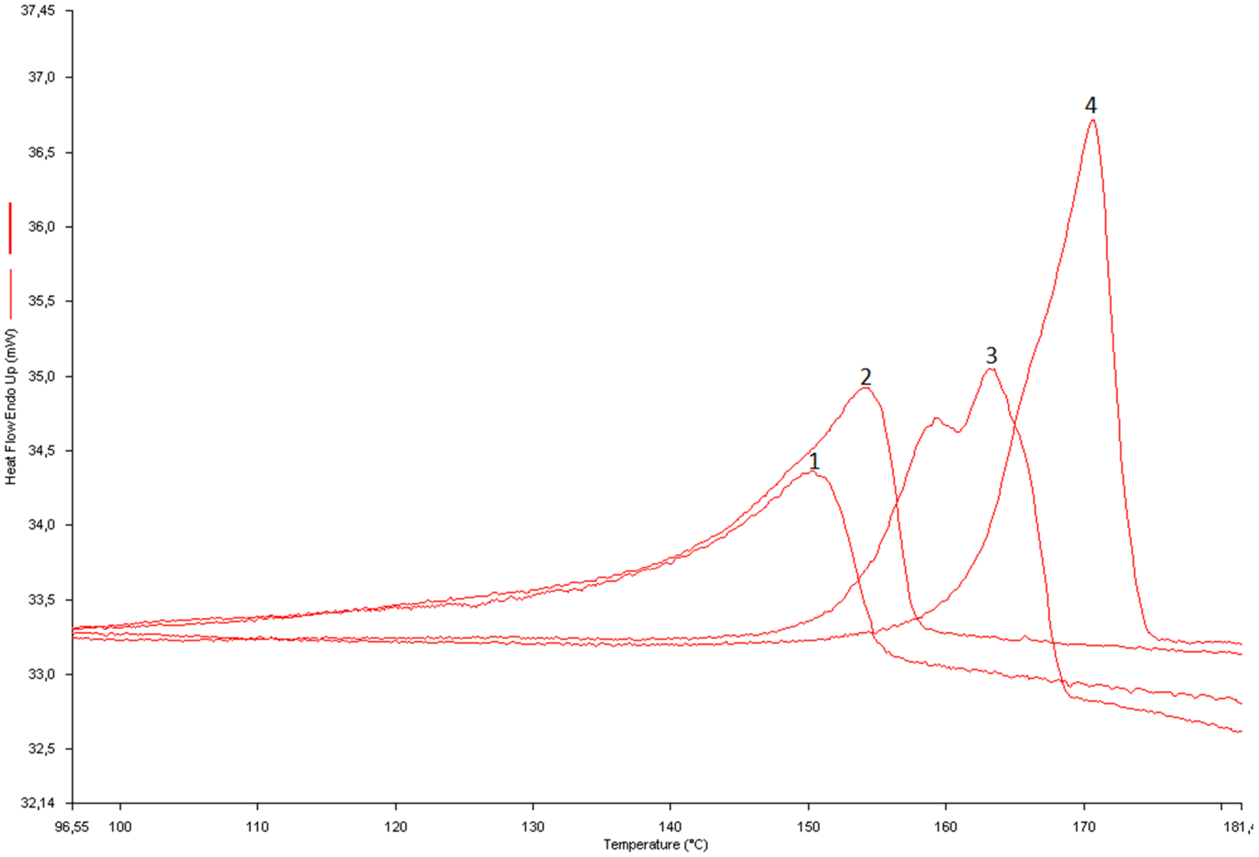

Figure S6. The results of minimum bactericidal concentration (MBC) determination for $Na_2Cu_3(CuOH)_2[W_{12}O_{40}(OH)_2]\cdot 32H_2O$ (μg/mL).

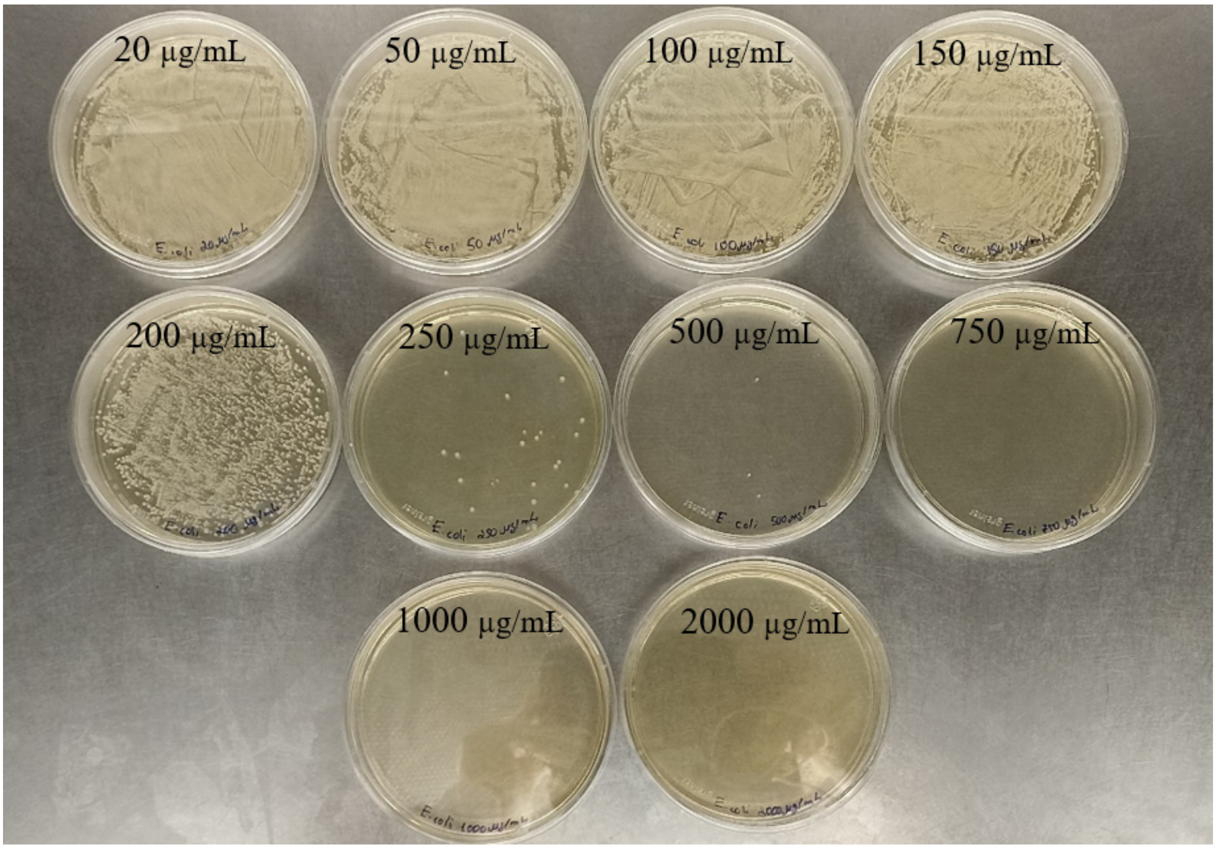

Figure S7. *E. coli* CFU after inoculation of the films in the wash by several dilutions (D – dilution factor): (a) Extr. PLA-POM film, (b) blank Extr. PLA film.

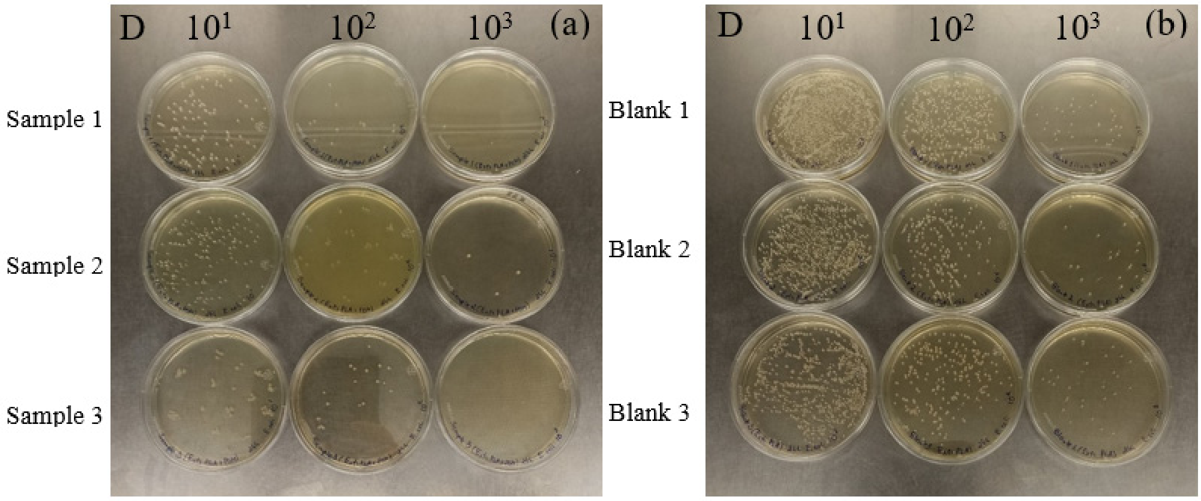

Figure S8. *E. coli* CFU after inoculation of the films in the wash by several dilutions (D – dilution factor): (a) PLA-POM film, (b) blank PLA film.

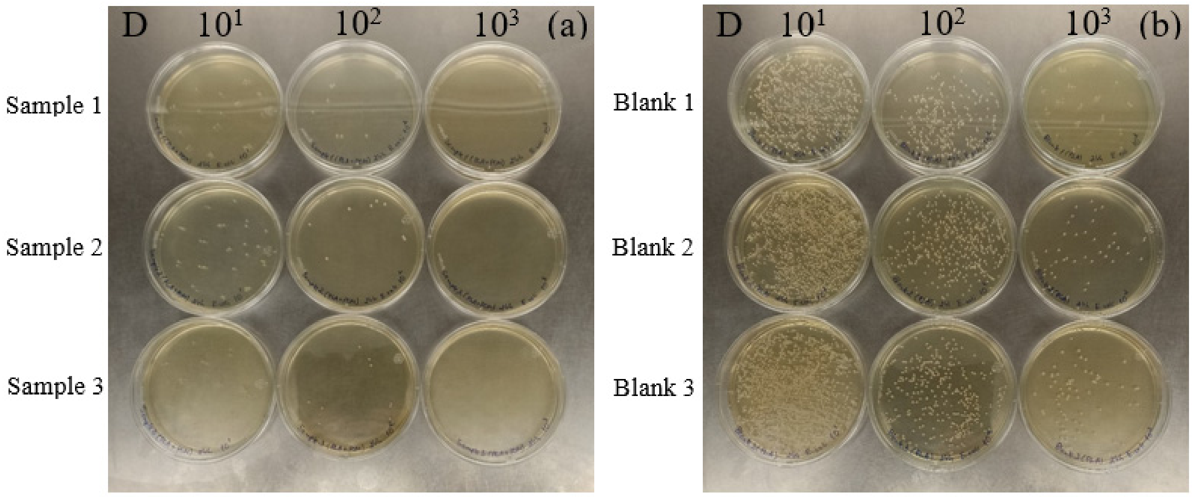

Supplement: Supplementary file 1 [file molecules-27-02510-s001.zip › molecules-1650764-supplementary.pdf]
